# Matrix-associated chondrocyte transplantation for reconstruction of articulating surfaces in the temporomandibular joint: a pilot study covering medium- and long-term outcomes of 6 patients

**DOI:** 10.1016/j.oooo.2018.02.017

**Published:** 2018-08

**Authors:** Gerhard Undt, Michael Jahl, Sebastian Pohl, Stefan Marlovits, Doris Moser, Hyang-Hee Yoon, Jimmy Frank, Susanna Lang, Christian Czerny, Guenter Klima, Eileen Gentleman, Rolf Ewers

**Affiliations:** a Department of Oral and Maxillofacial Surgery, Medical University of Vienna, Waehringer Guertel 18-20, 1090, Vienna, Austria; b Department of Trauma Surgery, Medical University of Vienna, Waehringer Guertel 18-20, 1090, Vienna, Austria; c Clinical Institute of Pathology, Medical University of Vienna, Waehringer Guertel 18-20, 1090, Vienna, Austria; d Department of Radiology and Nuclear Medicine, Medical University of Vienna, Waehringer Guertel 18-20, 1090, Vienna, Austria; e Clinical Institute of Pathology, Medical University of Innsbruck, Muellerstrasse 44, 6020 Innsbruck, Austria; f Centre for Craniofacial and Regenerative Biology, King's College London, London, SE1 9RT, United Kingdom

## Abstract

**Objective:**

Matrix-associated chondrocyte transplantation is routinely used in joints of the extremities but not in the temporomandibular joint (TMJ).

**Study Design:**

We report the first case series in 7 patients of a tissue engineering approach to regenerate severely degraded articulating surfaces in the TMJ by simultaneously completely resurfacing both the mandibular condyle and the articular eminence/glenoid fossa with a commercially available collagen sponge seeded with autologous cells stabilized within a fibrin matrix. To facilitate healing, we temporarily employed a silicone membrane to protect the engineered tissues. The indications for surgery were posttraumatic fibro-osseous ankylosis, ankylosing osteoarthritis, or late-stage osteoarthritis.

**Results:**

Six of the patients were recalled for follow-up after 3 years 6 months to 12 years 1 month. The maximum incisal opening was 18.2 ± 9.2 mm (range, 9-33 mm) before and 31.2 ± 13.6 mm (range, 12-47 mm) at the latest follow-up. Histologic specimens taken at 4 months showed beginning differentiation of fibrocytes into chondrocytes, whereas at 3 and 11 years, mature hyaline cartilage—not typical for the TMJ—was present.

**Conclusions:**

We conclude that the reconstruction of TMJ surfaces by matrix-associated chondrocyte transplantation may become a routine method for cartilage regeneration in the TMJ in the future.

**Statement of Clinical Relevance**

Seven patients underwent reconstruction of severely degraded articulating surfaces of the temporomandibular joint (TMJ) by a tissue engineering approach. Long-term results raise hope that the method could be applied routinely in patients who otherwise would be candidates for alloplastic TMJ replacement.

Alt-text: Unlabelled box

One important aim of the field of tissue engineering (TE) is to replace degenerated tissues with cells and scaffolds that restore tissue function and mediate regeneration. During the past decades, matrix-associated chondrocyte transplantation (MACT) has emerged as a disease-modifying treatment with excellent clinical long-term results in patients with isolated cartilage injuries and osteochondral lesions in the knee joint.^1^ The first report of this technique was published in 1994 by Brittberg et al.^2^ In the meantime, the MACT technique has been adapted for use in the ankle,^3^ hip,^4^ shoulder,^5^ and elbow joints^6^ with some success, but long-term follow-up studies are often lacking. Moreover, MACT has been reported to repair focal lesions, often on the patella or femoral condyle. TMJ degenerative disease, however, can affect the entire bearing surface and that on both the mandibular condyle and eminence/fossa simultaneously. To our knowledge, TE has not been attempted in humans to regenerate an entire articulating surface of a diarthrodial joint and has never been attempted on both bearing surfaces at the same time.

TMJ ankylosis in adults may result from injury, local or systemic infection, osteoarthritis, or systemic diseases such as rheumatoid arthritis or psoriasis. Also, multiple failed surgeries might result in ankylosis of the TMJ.^7^ It is a disabling condition that causes impairment in mastication, speech, and dental hygiene and, when affecting young individuals, also may cause deformity and asymmetry of the facial skeleton. Numerous procedures for the release of ankylosis and TMJ reconstruction have been described in the literature since 1850.^8^ Today, biological methods of TMJ reconstruction compete with alloplastic joint replacement. In children, the current preference is for autogenous reconstruction that can potentially grow with the child.^7^ Free and vascularized rib grafts^9^ or fibula free flaps^10^ are the methods most commonly used for biological reconstruction of the mandibular ramus. However, there is a high risk of re-ankylosis reported for these methods.^11^ Gap arthroplasty with interposition of autologous tissues is still a method of TMJ ankylosis treatment widely used today; temporalis muscle flaps,^9^ dermis-fat grafts,^12^ ear cartilage,^13^ or fascia lata^14^ are the most important methods of autogenous reconstruction. Although there have been reports about foreign-body reactions, alloplastic materials are still used for the permanent separation of free bone surfaces after gap arthroplasty.^15^ Alloplastic total joint reconstruction is the gold standard for treatment of TMJ re-ankylosis in the case of multiple failed previous surgeries or systemic disease.^7^ The potential disadvantages of alloplastic reconstruction relate to metal hypersensitivities and to degradation or failure of the material after more than 15 to 20 years.^16^ Also, a risk of 1.5% for early and late infections of alloplastic TMJ devices has been reported.^17^ Therefore, researchers and surgeons have been seeking new biological methods of TMJ reconstruction.^18^, ^19^ The first use of the MACT technique in the TMJs of patients with osteoarthritis was mentioned by Professor Michael Rasse (Wels, Austria, personal communication 2003); however, no reports thus far have appeared in the literature. Adopting an approach for tissue regeneration that has been successfully applied in other joints might be a step forward in biological TMJ reconstruction, and so we report here a case series of 7 patients treated with MACT to repair severely degraded articulating surfaces of the TMJ.

## Materials and Methods

### Patient population

From September 2003 to June 2009, we recruited 7 patients for TE-based TMJ reconstructions. Three patients were referred with posttraumatic fibro-osseous ankylosis, 3 patients with ankylosing osteoarthritis, and 1 patient with late-stage osteoarthritis and articular pain. Six of the patients had undergone previous TMJ surgeries, whereas one patient underwent the TE-based TMJ reconstruction as a first surgical procedure (Table I). One of the patients with posttraumatic ankylosis refused any further interventions after the MACT surgery and did not show up for subsequent appointments. Six of the 7 patients—5 females and 1 male, aged 27 to 66 years at the time of surgery—were recalled for follow-up after 3 years 6 months to 12 years 1 month.

**Table I:** Overview of patients

Characteristics	Patient 1	Patient 2	Patient 3	Patient 4	Patient 5	Patient 6	Patient 7
Gender	Male	Female	Female	Female	Female	Female	Female
Age at the time of MACT surgery, y	46	66	36	27	47	56	52
Operated side	Left	Bilateral	Bilateral	Right	Left	Left	Left
Underlying pathology	Posttraumatic ankylosis	Ankylosing osteoarthritis	Posttraumatic ankylosis	Ankylosing osteoarthritis	Severe osteoarthritis	Ankylosing osteoarthritis	Ankylosing osteoarthritis
Previous TMJ surgeries / orthognathic surgeries	2 / 0	1 left / 0	2 bilateral / 0	6 / 3	6 / 0	0 / 0	3/0
**Preoperative status**							
Articular pain	No	Yes / yes	Yes / yes	Yes	Yes	Yes	Yes
Muscle pain	No	Yes	Yes	Yes	Yes	Yes	Yes
Chronic pain	No	No	No	Yes	No	No	Yes
Maximum IID before surgery, mm	9	12	10	17	33 (limited by pain)	28	15
**MACT surgery**							
Date of MACT surgery	September 2003	November 2003	December 2003	September 2008	February 2009	June 2009	May 2009
Maximum IID gained during surgery, mm	32	43	45	48	Not measured	41	Not measured
**Removal of silicone sheet**							
Date of removal of silicone sheet	January 2004	March 2004	April 2004	January 2009	June 2009	November 2009	No silicone sheet inserted
Microscopic evaluation of silicone sheet	Tear, defect 1 × 1 mm	Tear, defect 1 × 1 mm left / tear right	Tear / tear + abrasion	Tear + abrasion	Abrasion	Abrasion	
Revision surgery performed	No	No	April 2014 (after 11 y)	October 2011 (after 3 y)	No	No	
Indication for revision surgery	None	None	Bilateral re-ankylosis	Pain	None	None	
**Long-term follow-up**							
Date of latest follow-up	September 2015	December 2015	December 2015 (April 2014)	March 2012	January 2015	November 2015	Lost to follow-up
Time of follow-up	12 y	12 y 1 mo	12 y	3 y 6 mo	6 y	6 y 5 mo	
Articular pain	No	No / no	No / no	Yes	No	No	
Muscle pain	No	Yes	Yes	Yes	Yes	No	
Chronic pain	No	No	No	Yes	No	No	
Malocclusion	No	No	No	No	No	No	
Maximum IID at latest follow-up, mm	26	37	38 (12 mm before revision)	18 (limited by pain)	47	47	
**Histology**							
Pre-existing fibrocartilage detected during MACT surgery	No	Yes / yes (highly degenerated)	Yes / yes	No	*~*	Yes (degenerated)	
Transition into fibrocartilage detected 4 mo after MACT surgery	Yes	No / no	Yes / yes	No	*~*	No	
Foreign bodies and reactive tissue 4 mo after MACT surgery	Yes	Yes (left) / no (right)	No / no	No	~	No	
Hyaline cartilage detected after MACT surgery	No	No	Yes / yes (2014, after 11 y)	Yes (2011, after 3 y)	~	No	

*MACT,* matrix-associated chondrocyte transplantation; *TMJ,* temporomandibular joint; *IID,* interincisal distance.

### Preoperative investigations and informed consent

**Fig. 1:**
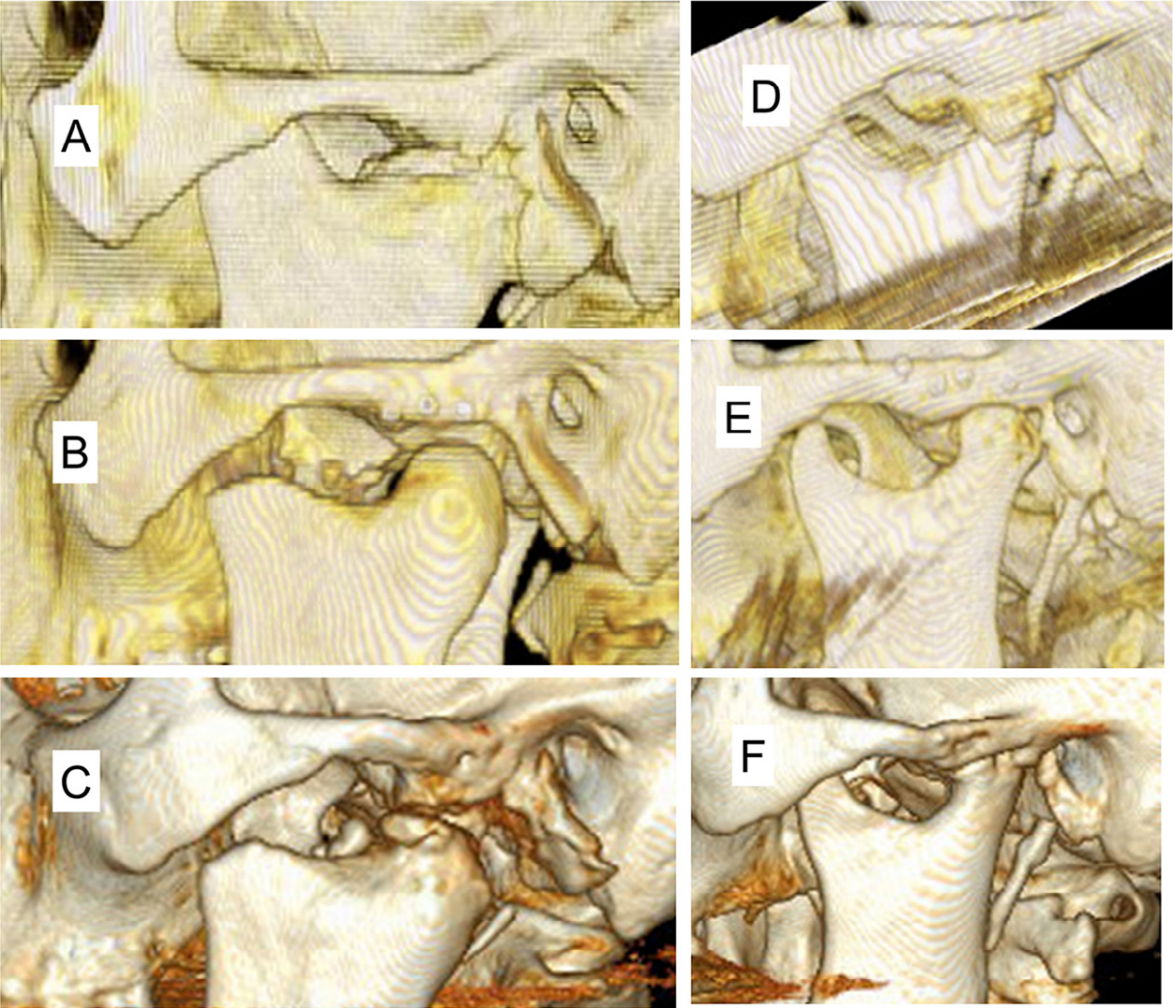
Three-dimensional computed tomography scans of patient 1 with posttraumatic ankylosis (**A-C**) and patient 2 with ankylosing osteoarthritis (**D-F**) preoperatively (**A** and **D**), postoperatively (**B** and **E**), and at 12-year follow-up (**C** and **F**).

All patients were examined clinically before surgery, and a detailed status of range of motion, occlusion, articular pain, and muscular pain was recorded (Table I). Maximum incisal opening (MIO) was measured between the tips of the upper and lower incisors at maximum unassisted opening. In addition, plain radiographs and a panoramic radiograph as well as a 3-dimensional computed tomography (CT) scan were recorded and the data sets processed for accurate preoperative planning (Figures 1A and 1D). All but one patient who suffered from claustrophobia also underwent a magnetic resonance imaging scan of the TMJ. Before undergoing the 3-step surgeries, each of the patients was informed about the planned procedures, alternative surgical methods available for treating their conditions, and possible intra- and postoperative complications. Each of the patients signed an informed consent for each component of the 3-step surgery.

### Autologous cartilage collection

Preparations for the extracorporeal augmentation of autologous chondrocytes in autologous patient serum were made according to the protocol of the Institute for Tissue and Organ Reconstruction (IGO, Wels, Austria). Autologous rib cartilage was harvested under general anesthesia. An incision about 4 cm long was made in the right submammary fold directly over the sixth rib, and the junction between bone and cartilage was exposed. After longitudinal incision of the perichondrium, 1 to 1.5 cm^3^ of cartilage was excised with a scalpel, and the chips were transferred to a transport container filled with a sterile transport medium (Ham's F-12 medium with L-glutamine, Lonza). After reassurance that no perforation of the pleura had occurred, the surgical wound was closed in layers. About 100 mL of venous blood was taken from the patient. Cartilage and blood were stored at room temperature and shipped to the laboratory in special transport containers. All patients tolerated the rib cartilage excision well.

### Extracorporeal in vitro cell augmentation

After a maximum time of 48 to 72 hours in the transport medium, primary autologous chondrocyte cultures were established. The cartilage segments were washed twice in Hank's balanced salt solution (Gibco, Paisley, Scotland) and incubated for 1 hour in a 0.05% trypsin solution (Gibco). The tissue was then mechanically sliced into pieces approximately 1 mm^2^ in size. These were subsequently incubated in a solution of 0.1% collagenase CLS II (Worthington Biochemical, Freehold, NJ) in phosphate-buffered saline without Ca^2+^ and Mg^2+^ (Gibco) and shaken in Erlenmeyer tubes (Corning Glass Works, Corning, NY) for 24 hours at 37°C in a shaking water bath. After incubation, the suspension was filtered through a 100-µm nylon cell strainer (Falcon, Franklin Lakes, NJ) and centrifuged. The pellet was resuspended in Medium-199 (Gibco), which contained 10 ng/mL basic fibroblast growth factor (Boehringer Ingelheim, Germany) and 10% autologous patient serum. The cells were then plated on T12 culture flasks (Falcon) precoated with fibronectin (Sigma, St. Louis, MO). The cells adhered to the flask surface, spread, and proliferated as a monolayer culture. Half the culture medium was changed every second day. After confluency, the cultures were transferred to T75 culture flasks.^20^ Cells were cultured for approximately 4 weeks, and the initial 100,000 to 300,000 cells isolated from the tissue were expanded into approximately 20 million to 120 million cells.

### Ankylosis surgery and reconstruction by MACT

**Fig. 2:**
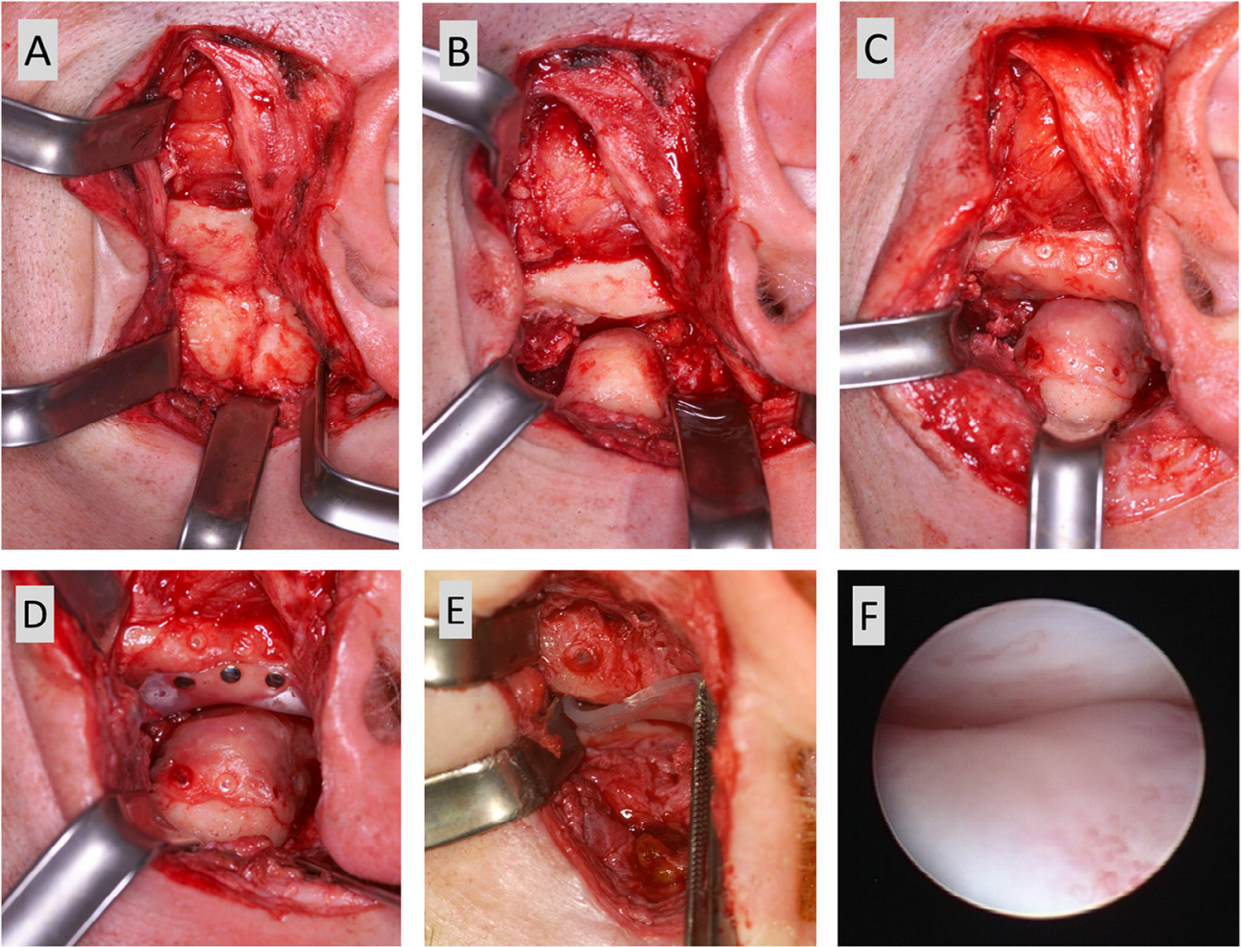
Patient 1. (**A**) Posttraumatic ankylosis of left temporomandibular joint. (**B**) After lysis of ankylosis, a gap of about 5 mm is created. (**C**) The skull base and the condylar head have been lined with collagen scaffolds seeded with chondrocytes. They are attached to the bone with resorbable polylactide pins. (**D**) A 1-mm silicone sheet is interposed and attached to the lateral glenoid fossa with titanium pins. (**E**) Removal of the silicone sheet after 4 months. (**F**) View of the joint space after the removal of the sheet. Vascularized tissue covering the condylar head and the skull base is visualized.

**Fig. 3:**
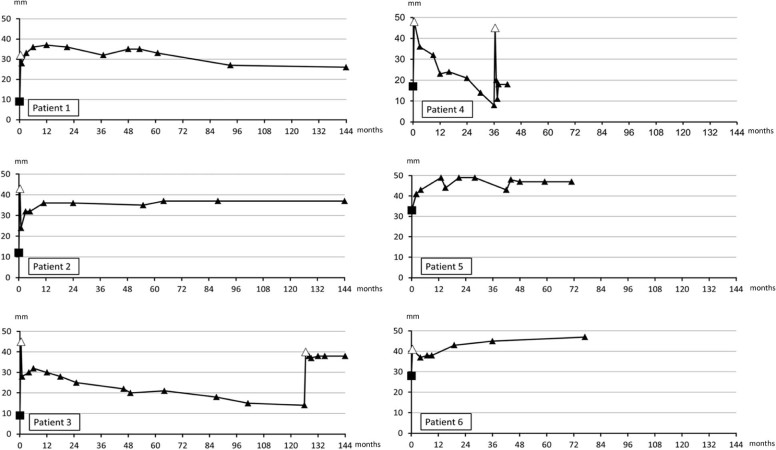
Maximum incisal opening (MIO) in mm of patients 1-6 followed up to 144 months. The black square (■) indicates preoperative MIO, the white triangle (Δ) indicates maximum forced intraoperative MIO, and the black triangles (▴) indicate MIO at different follow-up times.

Under general anesthesia with transnasal intubation and under antibiotic coverage, the ankylosed TMJ was opened by the same approach that had been used during the preceding surgeries. In cases in which the patient had previously been operated on at our clinic or had the first ankylosis surgery, we used our standard approach to the TMJ.^21^ The lateral aspect of the TMJ was exposed (Figure 2A), and lateral bone masses were removed with the aid of straight and curved chisels. This technique allowed for the removal of large masses of bone without creating bone dust that might have been a source of re-ankylosis.^22^ The fibrous gap between the bone masses covering the skull base and the condylar head was localized, and the bony surfaces of the skull base and the condylar head were contoured with chisels but also with rotating burs and reciprocating bone rasps, if necessary (Figure 2B). When these instruments were used, the bone dust was removed meticulously by irrigation and suction. If bone close to the middle cranial fossa and in medial parts of the joint had to be removed, we used a Piezosurgery device (Mectron Dental, Germany) that allowed bone resection with a reduced risk of damage to adjacent structures such as the dura of the parietal lobe, the middle ear, or the middle meningeal artery. In instances of poor vision of the medial areas of the joint, we used a 1.9-mm-diameter, 0° rod-lens arthroscope (Henke-Sass, Wolf, Tuttlingen, Germany) in connection with an Olympus endoscopy tower to gain a magnified view of critical structures. The removed bone and fibrous tissue were collected for histologic examination and stored in 4.5% formalin. In contrast to the traditional technique of creating a wide gap of about 2 cm between the ramus and the fossa, we contoured the surfaces carefully in congruency to each other just leaving a small gap of about 5 mm that allowed interposition of the chondrocyte-containing scaffolds. By maintaining the full height of the mandibular ramus, the occlusion of the patients should not be altered. After resection of the ankylosis, the maximum interincisal distance that could be achieved by use of a Roser-Koenig mouth gag between the molars of the patient was photodocumented. The intraoperatively measured values for MIO are displayed in Table I and Figure 3.

**Fig. 4:**
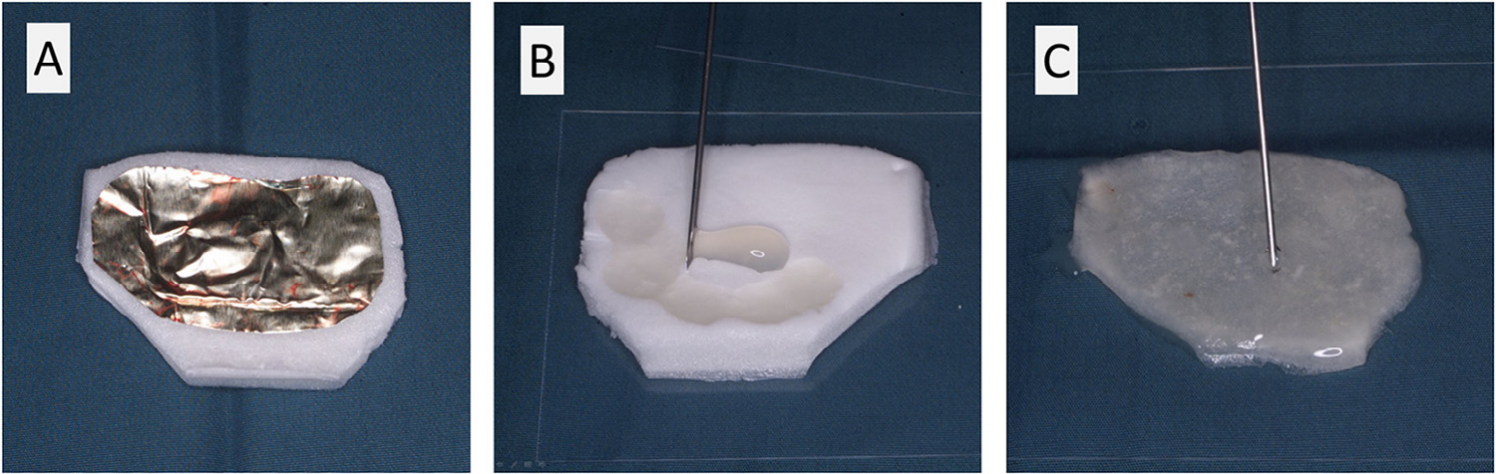
Preparation of the collagen scaffold. (**A**) The scaffold is cut according to the shape of a template representing the size of the area that should be covered with cartilage. (**B**) Chondrocytes dispersed in stabilized fibrinogen are spread to both sides of the scaffold. (**C**) The construct is stabilized by spreading stabilized thrombin solution to its surfaces.

The next step was outlining the size of the areas on the skull base and condylar head that should be covered with the chondrocyte-seeded scaffold. Metal sheets tailored out of the packaging of suturing materials were trimmed to the desired shape, and equine collagen scaffolds (TissuFleece E, 7 × 3 cm, Resorba, Nürnberg, Germany, distributed by Baxter, Vienna, Austria) were tailored accordingly (Figure 4A). Then the pellet of chondrocytes was dispersed in a stabilized fibrinogen solution provided by IGO and spread on the two scaffolds with the aid of a syringe and an 18-G cannula. Stabilization of the commercially available fibrin glue (Immuno, Vienna, Austria) was achieved by adding 8500-IE/mL aprotinin (Bayer, Leverkusen, Germany) and 15 mg/mL tranexamic acid (Pharmacia, Stockholm, Sweden) to the solution. The collagen scaffolds were soaked with the cell suspension, and the construct was stabilized by adding the second component of the fibrin glue, a stabilized thrombin solution (Figures 4B and 4C). After reaction of the two components, the construct was transferred to the joint. First the articulating surface of the skull base was covered, and the construct was fixed to the zygomatic arch and the articular eminence with the aid of resorbable membrane nails (Resor-Pin, Geistlich Biomaterials, Wolhusen, Switzerland) or with Sonic Weld pins (KLS Martin Co., Tuttlingen, Germany) (Figure 2C). Then the construct on the skull base was secured by a 1-mm-thick silicone sheet (Bess Rhino silicone sheeting unrestricted 50 × 70 × 1 mm, Bess Medizintechnik, Berlin, Germany) that also had been tailored to fit into the joint gap. We fixed this sheet with titanium nails (Frios Dentsply, Germany) firmly to the zygomatic arch (Figure 2D). The rationale for temporarily interpositioning this alloplastic material was to prevent shearing of the scaffolds from the bone surface and allow immediate load and function. The last step of the MACT procedure was covering the condylar head with a cell-seeded collagen matrix and fixing this matrix to the anterior, lateral, and posterior aspects of the condylar head. Then the surgical wound was closed in layers, leaving the tip of a small-sized drain close to the joint. This drain was removed the following day. Intensive physiotherapy started between the third and the seventh day after surgery. No postoperative surgical complications were observed in any of the patients.

### Removal of the silicone sheet 4-5 months after the MACT surgery and assessment of early repair

The third step of the procedure was carried out under general anesthesia. The joint was approached by the same incision as before, and the titanium nails and the silicone sheet were removed. The new joint space that corresponded exactly to the extent of the sheet was explored with the 1.9-mm arthroscope, and joint function was confirmed (Figures 2E and 2F). Histologic specimens of the soft tissues and microtrephine bone biopsies in lateral parts of the joint were gained under arthroscopic guidance and stored in 4.5% formalin. If function was impaired by remaining bone or adhesions, we corrected these pathologies before closing the wound in layers. Intensive physiotherapy in combination with daily exercises at home continued after a short intermission postsurgery.

### Histologic preparation

**Fig. 5:**
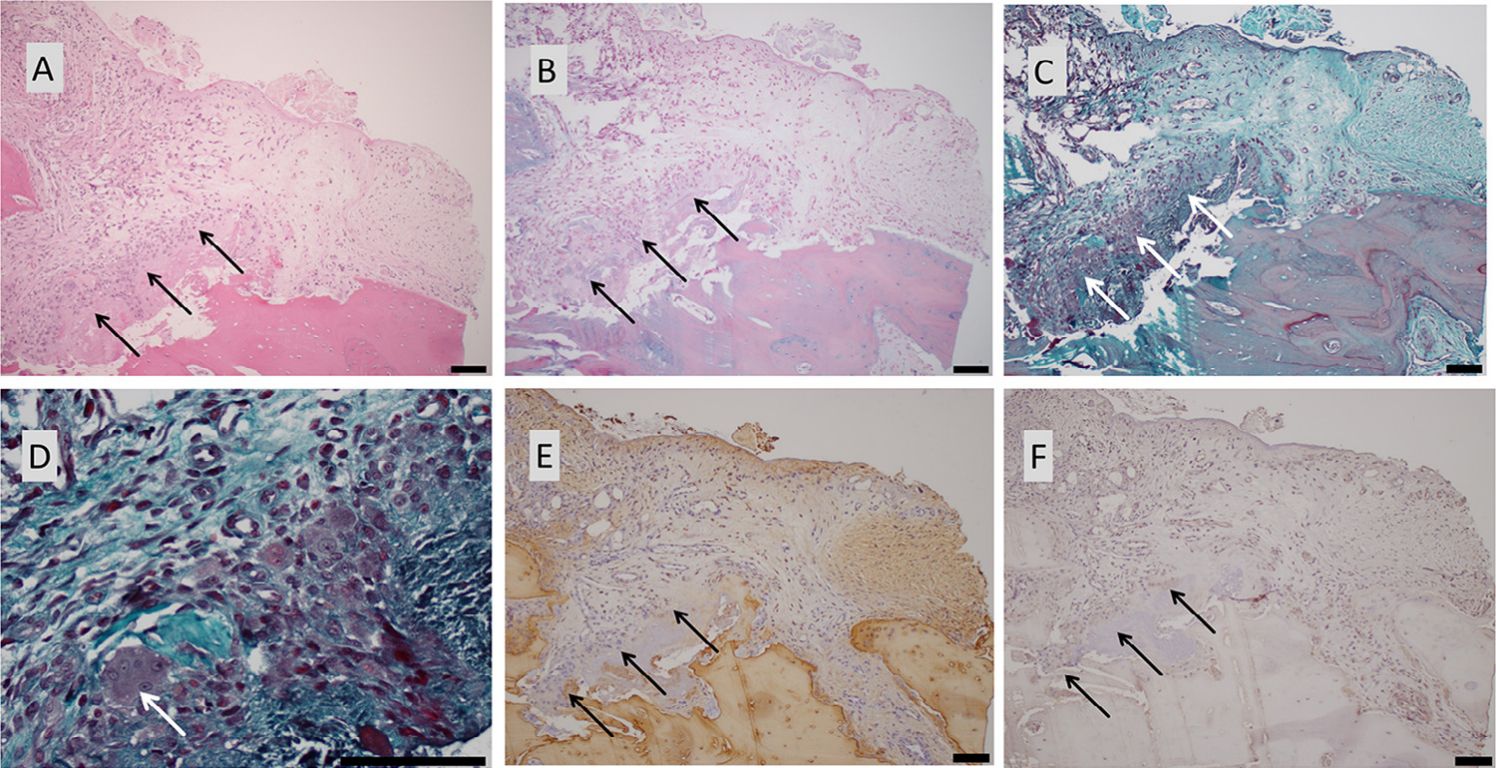
Histology of a microtrephine specimen from the lateral glenoid fossa 4 months after matrix-associated chondrocyte transplantation in patient 1. (**A**) Hematoxylin and eosin staining 10 × , (**B**) alcian blue staining 10 × , (**C**) safranin-O staining 10 × , (**D**) safranin-O staining 40 × , (**E**) collagen type I staining 10 × , and (**F**) collagen type II staining 10 × . The arrows indicate the zone of transformation of fibrocytes into chondrocyte-like cells.

Specimens were stored in a 4.5% formalin solution before further processing. Bony specimens were first decalcified in an ethylenediaminetetraacetic acid solution for 12 to 36 hours. All specimens were then dehydrated overnight and embedded in paraffin blocks. These blocks were sectioned to slices about 3 µm thick. The specimens were stained with hematoxylin and eosin (HE) routinely. Safranin-O and alcian blue staining procedures were used for the detection of cartilage matrix in samples of engineered tissues. Immunohistochemical staining for collagen type I and collagen type II was also performed in selected specimens (Figure 5).

## Results

### Clinical outcome and radiologic results

One patient with posttraumatic ankylosis was lost to follow-up after the procedure. The remaining 6 patients were followed up with between 3 years 6 months and 12 years 1 month. The most important clinical criterion for assessing a favorable outcome in patients who undergo TMJ reconstruction is a good range of mouth opening. Mean MIO in our group of patients was 18.2 ± 9.2 mm (range, 9-33 mm) before and 31.2 ± 13.6 mm (range, 12-47 mm) at the latest follow-up (Table I; Figure 3).

Two of the six patients underwent revision surgery. Their history and clinical follow-up is presented in detail.

Patient 3, female, had bilateral condylar fractures in 2002 and underwent bilateral open reduction and osteosynthesis. Because of persisting pain and restricted motion, a revision surgery was performed at another hospital, which resulted in bilateral fibro-osseous ankyloses. In 2003, bilateral lysis of ankylosis and MACT reconstructions were performed. When the silicone membranes were removed bilaterally 4 months after operation, heterotopic bone formation was detected on both sides and a bony bridge from the left zygomatic arch to the condylar head had developed over the silicone sheet. Another lysis of ankylosis was performed, and the patient was referred to bilateral radiotherapy. Ten Gy were applied to each side, and the patient maintained an MIO of >25 mm for 2 years (Figure 3). When her MIO went down to 20 mm 4 years after MACT surgery (2007), we proposed an alloplastic reconstruction of both joints. The patient waited until 2014, when her MIO was restricted to 12 mm, and decided to have another lysis of ankylosis and autologous fat grafts instead of an alloplastic reconstruction. Her mouth opening at the latest follow-up in December 2015 was stable at 38 mm for more than 1 year.

**Fig. 6:**
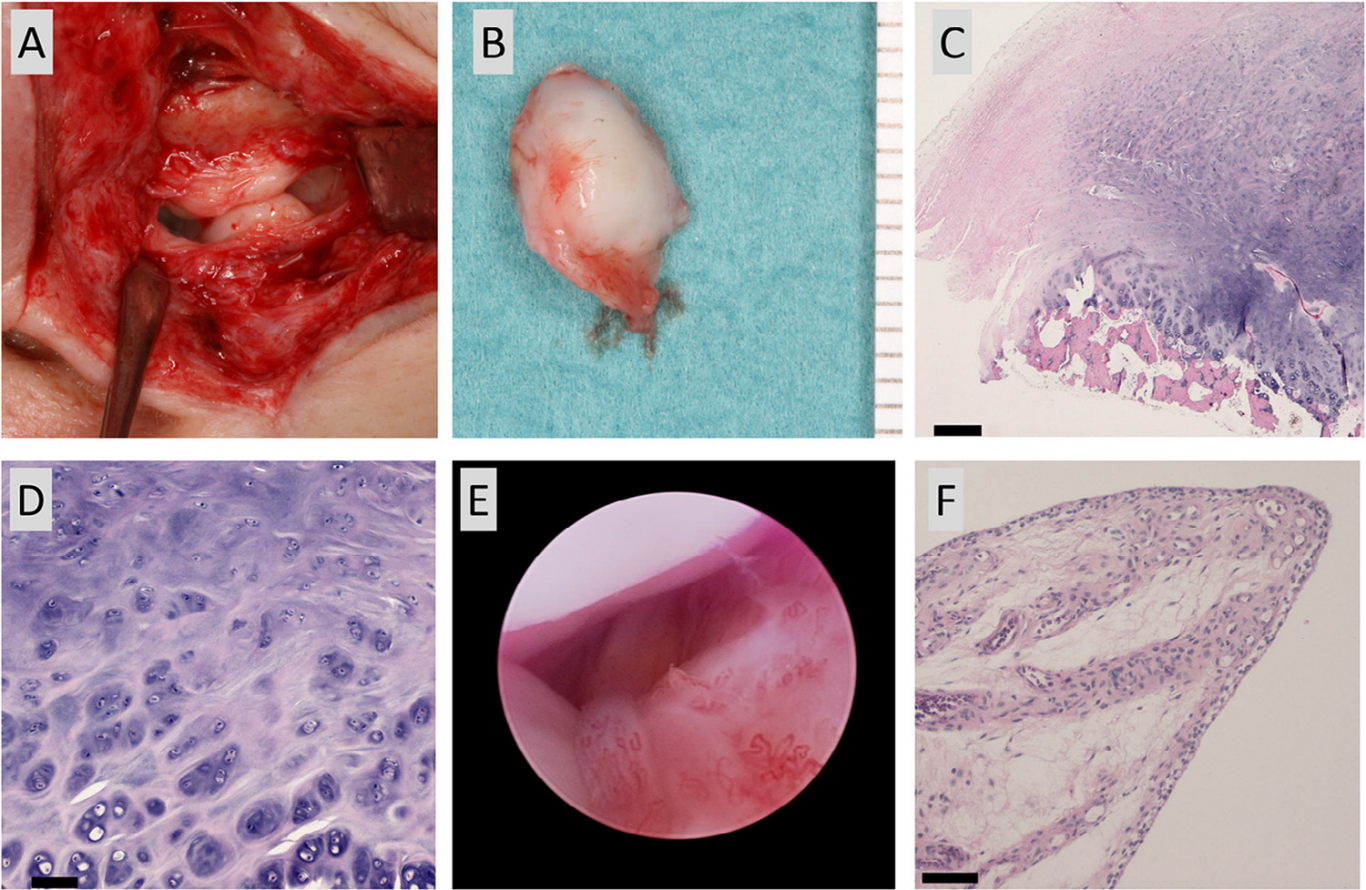
Second-look surgery in patient 4 three years after matrix-associated chondrocyte transplantation. (**A**) Intraoperative view of the right temporomandibular joint showing thick cartilage covering of the glenoid fossa, the articular eminence, and the condylar head. The condyle is held in its maximal anterior position by manually guided forward movement of the mandible. The posterior and the anterior joint recesses are opened and show that a new joint capsule with synovial lining has formed. (**B**) Lateral osteophyte of the condylar head, removed with a chisel. (**C**) Histologic specimen of the osteophyte, hematoxylin and eosin (HE) staining 5 × . (**D**) HE staining 40 × . The specimen shows hyaline cartilage. (**E**) Polypous synovitis in the anterior joint recess. (**F**) Section through the tip of a synovial polyp (HE staining 20×).

Patient 4, female, had undergone six TMJ surgeries and three orthognathic surgeries elsewhere when she presented with a fibro-osseous ankylosis, joint pain, muscular pain, and a chronic pain component. She was informed that she should not expect any improvement of the chronic pain component and that the benefit of the surgery would be better mouth opening. After lysis of ankylosis and MACT reconstruction in 2008, she gained an MIO of 36 mm, which decreased to 21 mm after 1 year and to 8 mm after 3 years. This limitation was caused primarily by articular and muscular pain, and still the chronic pain component was not treated properly with analgesic medication. During a second-look surgery in 2012, her mouth could be opened easily during intubation. All the joint surfaces were covered by new cartilage, and perfect translation of the condylar head could be demonstrated by moving the mandible forward and backward (Figure 6A). Tissue samples were taken from a lateral cartilage-covered osteophyte and from a synovial polyp before the application of electrocoagulation (Figures 6B-6D). The cause for her articular pain component was found with the aid of an arthroscope: pronounced polypus synovitis of the new synovial lining of the anterior joint wall and creeping synovitis on marginal areas of the new cartilage (Figures 6E and 6F). These inflamed tissues were cauterized under arthroscopic view with a diathermy probe. Maximum intraoperative mouth opening was 45 mm. Although the intraoperative findings had shown perfect joint structures, the patient could not open her mouth more than 18 mm after the operation because of severe pain. She sought treatment at another hospital and underwent alloplastic TMJ reconstruction.

### Histologic results

**Fig. 7:**
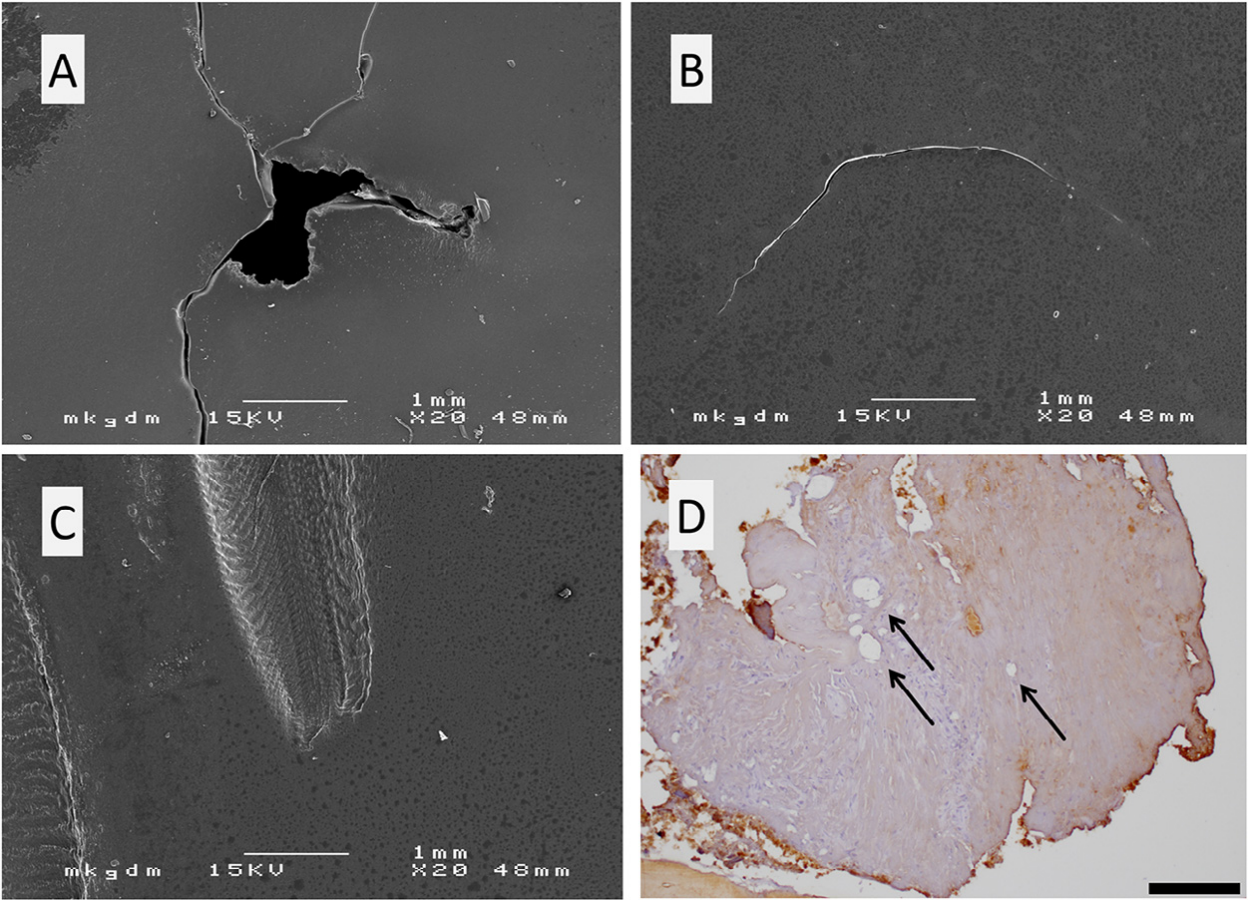
Signs of wear in the silicone membranes detected by the scanning electron microscope. (**A**) Defect of 1 × 1 mm (patient 1), (**B**) tear (patient 3), (**C**) abrasion (patient 5), and (**D**) biopsy from regenerated tissue 4 months after matrix-associated chondrocyte transplantation (patient 2). Collagen type I staining 10 × . The arrows indicate silicone particles with mild foreign-body reaction.

In 3 of the 7 joints where MACT was applied for reconstruction after ankylosis, fibrocytes with signs of differentiation to chondrocyte-like cells could be detected 4 months after surgery when the silicone sheet was removed (Table I). Figure 5 shows a microtrephine specimen of lateral parts of the glenoid fossa obtained from patient 1 at 10 × magnification. Close to the underlying bone, there is a zone of fibrous tissue, where rounded, chondrocyte-like cells are evident (Figure 5A, HE staining 10 × , arrows). Safranin-O 10 × and 40 × and alcian blue staining show the presence of glycosaminoglycans, a major component of extracellular cartilage matrix, around these cells (Figures 5B-5D). Immunohistochemical staining for collagen type I and collagen type II revealed the presence of abundant collagen type I in these specimens (Figures 5E and 5F). Together, these data suggest that the TE-based reconstruction was able to initiate repair of the joints' articulating surfaces. Figure 6 displays histologic specimens from patient 4 three years after MACT in the right TMJ. A section through a lateral osteophyte (Figures 6C and 6D, magnification 5 × and 40×) shows a thick layer of hyaline cartilage, a tissue that is not observed in the TMJ under regular conditions. Figure 6F represents a section through the tip of an inflamed synovial polyp in the anterior recess of the joint. It shows vascularized connective tissue lined by intact synovial tissue. For patients 1 and 2, whose silicone membranes appeared macroscopically to have undergone the most extensive wear and showed small perforations under the scanning electron microscope, histologic assessments of biopsies were notable for round ghost-like structures with diameters between 5 and 50 µm, suggestive of silicone particles present in the tissue, and a mild foreign-body reaction as indicated by the presence of giant cells (Figure 7D). No foreign-body reaction or “ghosts” were detected in biopsies from patients whose silicone membranes showed only surface tears or abrasions.

### Scanning electron microscope findings

The silicone sheets that were removed after 4 months were washed in distilled water and air-dried. The regions of interest were defined with the aid of a stereo microscope (SMZ 1500, Nikon, Japan), cut out, fixed on specimen mounts, sputter-coated with gold (Sputter Coater SC 502, Polaron, Fison Instruments, England), and then examined in a scanning electron microscope (JSM 6310, Jeol Ltd., Japan) at an acceleration voltage of 15 kV. The results are shown in Table I and Figure 7. Small perforations with a maximum size of 1 × 1 mm (Figure 7A), tears (Figure 7B), and abrasions (Figure 7C) were found.

## Discussion

Here we describe the first case series in which degenerated articular surfaces of the TMJ were repaired to enhance function and prevent re-ankylosis using a TE-based strategy. Four of our 6 patients showed excellent outcomes 6 to 12 years after reconstruction as measured by MIO. Two of our patients went on to have revision surgeries; however, the time between surgeries appears to have been extended based on the patients' previous presentations.

Surgical interventions to repair cartilage lesions using MACT have been described. These procedures aim to repair focal cartilaginous lesions, as they rely on mechanical support and healing from the surrounding native cartilage. Our approach here was to instead reconstruct both full articulating surfaces by stabilizing autologous chondrocytes within a collagen scaffold. We protected the TE surfaces by temporarily nailing a silicone sheet to the eminence/fossa to prevent potentially shearing the reconstruction before regeneration had taken place. Although we deemed this strategy to be successful overall, we did observe damage to the silicone membranes and structures indicative of the presence of silicone particulate matter in the repaired tissue. This would suggest that a more robust material with better wear resistance would be preferable in future repairs.

The medium- and long-term results of our small series of patients reconstructed by MACT suggest that in case of ankylosis it might not be necessary to create a gap of at least 1.5 to 2 cm between the mandibular ramus and the glenoid fossa in order to prevent re-ankylosis as proposed by Kaban et al.^23^ To respect this distance is also the argument for surgeons advocating the use of alloplastic TMJ devices, but even after total joint replacement with the currently used systems, heterotopic bone formation bridging the gap between the skull base and the ramus has been observed.^24^ We know from the literature that gap arthroplasty alone and gap arthroplasty with interposition of various materials may lead to a high rate of postoperative malocclusions. Bhatt et al.^25^ presented a series of 207 patients with gap arthroplasty and 55 patients with interposition arthroplasty. After a mean follow-up of 3.8 years, open bite was present in 9.2% of patients in the gap arthroplasty group and in 34.5% of the interposition arthroplasty group. Also, the rate of re-ankylosis in this collective, especially in patients who had undergone previous surgeries before, was very high: The recurrence rate in multiply operated patients was 34.5% in the patient group after gap arthroplasty and 30.8% in the group after interposition arthroplasty, whereas the recurrence rates in first-time operated patients was 14.7% and 4.8%, respectively. If the resected condyle is replaced by a free rib graft as described by Kaban in 1990,^26^ the occlusion seems to remain stable at least during a period of 1 year, but overgrowth or atrophy of the rib graft occurs in up to 50% of the cases, leading to facial asymmetry.^27^ The use of free rib grafts for TMJ reconstruction also appears to have a high rate of re-ankylosis as reported by Saeed and Kent in 2003.^11^

Babu et al., in a prospective study in 2013,^28^ have shown that aggressive gap arthroplasty might not be necessary for successful treatment of ankylosis. Fifteen patients (18 joints) with ankylosis of the TMJ were treated, all by creating a minimal gap with a vertical distance of 5 to 8 mm and by interposition of temporal fascia. Thirteen of the cases were posttraumatic, and in 2 cases, the etiology was infection. The mean age of the patients was 20 ± 8 years (range, 7-29 years), and the mean MIO before surgery was 3.9 ± 2.6 mm (range, 0-9 mm). The patients were followed for a minimum of 3 years. At follow-up, the mean MIO was 34.2 ± 3.1 mm (range, 30-40 mm). No recurrence was observed, and malocclusions could be managed by orthodontics.

Some surgeons consider alloplastic TMJ reconstruction as the current “gold standard” treatment for TMJ ankylosis. In 2002, Mercuri et al.^29^ reviewed the outcomes of 58 of 215 patients who had been implanted with Techmedica (TMJ Concepts) System devices between 1990 and 1994. These patients had undergone a mean of 4.2 ± 2.9 unsuccessful prior TMJ operations (range, 0-12 unsuccessful prior TMJ operations). The mean follow-up time was 107.4 ± 15.5 months (range, 60-120 months). Our patients had undergone 2.8 ± 2.3 unsuccessful prior surgeries (range, 0-6 unsuccessful prior surgeries), and the mean follow-up time of our patients was 100.7 ± 39.2 months (range, 42-144 months). In the patients after alloplastic TMJ reconstruction, a preoperative mean MIO of 25.5 ± 11.4 mm (range, 1-45 mm) and a mean MIO at follow-up of 33.2 ± 9.7 mm (range, 12-55 mm) was reported. In our patients, mean MIO before surgery was 18.2 ± 9.2 mm (range, 9-33 mm) and 31.2 ± 13.6 mm (range, 12-47 mm) at the latest follow-up. These data show that the outcomes for both patient groups are comparable and that our approach to TMJ reconstruction may give similar results to alloplastic reconstruction.

In our study, we used a silicone sheet for temporary separation of the articulating surfaces. Several studies have described foreign-body reactions in response to perforations of the material,^30^ and in 2 of our patients who developed single perforations with a size of about 1 × 1 mm, silicone particles were found in the tissue biopsies at the time of removal of the sheets. Unfortunately, there is no other material today with similar properties—in terms of elasticity, biocompatibility, and a hydrophobic surface—that allows that maintenance of a well-defined space until the protected tissues have stabilized and the sheet can be removed. Future developments in the field of biocompatible materials could bring more durable or degradable materials with similar properties that could be left in place and potentially change the current 3-step procedure into a two-step procedure. Promising materials seem to be spider silk constructs.^31^

For our approach, we harvested autologous chondrocytes from rib cartilage. The use of rib cartilage grafts is well-established in maxillofacial surgery; however, rib cartilage is hyaline while the TMJ articular surface is fibrocartilaginous. In vitro studies have suggested that chondrocytes derived from hyaline cartilage are superior to those isolated from the TMJ in terms of extracellular matrix secretion. Wang et al.^32^ compared the properties of cultivated cells derived from porcine TMJ chondrocytes with cells cultured from porcine ankle chondrocytes. Anderson and Athanasiou^18^ did the same with goat TMJ chondrocytes and rib cartilage chondrocytes. Both teams came to the conclusion that hyaline cartilage cells from other parts of the body appear better suited for TE fibrocartilage of the TMJ than chondrocyte-like cells harvested from TMJ fibrocartilage because of the high quantity of collagen and glycosaminoglycans and the better tensile and compressive mechanical properties of hyaline cartilage. Biopsies collected from the TMJs of our patients 4 to 5 months postreconstruction showed good early repair. Nevertheless, in second-look biopsies from patients 3 and 4, 11 and 3 years after TE reconstruction, respectively, we observed a thick layer of hyaline cartilage covering the bony surface, suggesting that the rib chondrocytes (although extensively expanded in vitro) may have mediated a hyaline-like, rather than a more physiologic fibrocartilaginous, repair. As we do not have second-look biopsies from all patients, it is unclear whether similar repairs also occurred in patients with successful outcomes.

Salash et al.^19^ recently outlined considerations for TE of the TMJ. They suggested that underlying pathologic processes that lead to TMJ deterioration will also attack engineered tissues, and therefore a TE approach to treat ankylosis and osteoarthritis is contraindicated. Although this may be true in the knee where MACT has produced mixed results for osteoarthrosis patients,^33^ our case series shows that regeneration can be successful in patients with TMJ ankylosis of both traumatic and osteoarthritic origin.

One possible application of TE in the TMJ was outlined in the article by Salash et al.^19^ As the replacement of the articular disc does not seem to be feasible at the current state of TE, lining the articular fossa with resistant engineered cartilage tissue would be an alternative in patients after discectomy. This tissue would serve as an interpositional buffer between the eminence and the condyle and promote joint congruence while improving mandibular function. In our opinion, MACT could be one way to create such a “fossa liner.”

In conclusion, our case series of 7 patients treated with a TE-based approach to correct TMJ ankylosis and severe osteoarthritis relieved pain and prevented re-ankylosis in the mid- and long-term in at least 4 patients. By using this technique, the height of the mandibular ramus was preserved and malocclusion could be avoided. Formations of hyaline cartilage and new synovial tissue have been found in 3 joints of 2 patients after 3 and 11 years. The relatively high failure rate of about one-third in our collective could be avoided in the future by excluding patients with a chronic pain component and patients with multiple previous operations and with extreme heterotopic bone formation.
